# Downregulation of Endothelial Plexin A4 Under Inflammatory Conditions Impairs Vascular Integrity

**DOI:** 10.3389/fcvm.2021.633609

**Published:** 2021-05-04

**Authors:** Dianne Vreeken, Caroline Suzanne Bruikman, Wendy Stam, Stefan Martinus Leonardus Cox, Zsófia Nagy, Huayu Zhang, Rudmer Johannes Postma, Anton Jan van Zonneveld, Gerard Kornelis Hovingh, Janine Maria van Gils

**Affiliations:** ^1^Department of Internal Medicine (Nephrology) and the Einthoven Laboratory for Vascular and Regenerative Medicine, Leiden University Medical Center, Leiden, Netherlands; ^2^Amsterdam Cardiovascular Sciences, Department of Vascular Medicine, Amsterdam UMC, Amsterdam, Netherlands; ^3^Novo Nordisk A/S, Copenhagen, Denmark

**Keywords:** neuroimmune guidance cues, plexin, semaphorin, inflammation, endothelial function, atherosclerosis

## Abstract

**Objective:** Besides hyperlipidemia, inflammation is an important determinant in the initiation and the progression of atherosclerosis. As Neuroimmune Guidance Cues (NGCs) are emerging as regulators of atherosclerosis, we set out to investigate the expression and function of inflammation-regulated NGCs.

**Methods and results:** NGC expression in human monocytes and endothelial cells was assessed using a publicly available RNA dataset. Next, the mRNA levels of expressed NGCs were analyzed in primary human monocytes and endothelial cells after stimulation with IL1β or TNFα. Upon stimulation a total of 14 and 19 NGCs in monocytes and endothelial cells, respectively, were differentially expressed. Since plexin A4 (PLXNA4) was strongly downregulated in endothelial cells under inflammatory conditions, the role of PLXNA4 in endothelial function was investigated. Knockdown of PLXNA4 in endothelial cells markedly impaired the integrity of the monolayer leading to more elongated cells with an inflammatory phenotype. In addition, these cells showed an increase in actin stress fibers and decreased cell-cell junctions. Functional assays revealed decreased barrier function and capillary network formation of the endothelial cells, while vascular leakage and trans-endothelial migration of monocytes was increased.

**Conclusion:** The current study demonstrates that pro-inflammatory conditions result in differential expression of NGCs in endothelial cells and monocytes, both culprit cell types in atherosclerosis. Specifically, endothelial PLXNA4 is reduced upon inflammation, while PLXNA4 maintains endothelial barrier function thereby preventing vascular leakage of fluids as well as cells. Taken together, PLXNA4 may well have a causal role in atherogenesis that deserves further investigation.

## Introduction

Atherosclerosis is a slow progressing pathophysiological process that ultimately leads to overt clinical manifestations of cardiovascular disease (CVD) (1), which remains the leading cause of death worldwide (2). Although the role of dyslipidemia in the development of atherosclerosis is widely acknowledged, half of the patients suffering from a myocardial infarction have normal lipid levels (3). It has become increasingly apparent that hyperlipidemia is not the sole driver of atherogenesis. Epidemiological, genetic association and intervention studies have shown that inflammation is an important determinant in the initiation and the progression of atherosclerosis as well (1, 4). Both interleukin 1β (IL1β) and tumor necrosis factor α (TNFα) have been shown to play a critical role in atherogenesis, *in vitro* as well as *in vivo* (5, 6). The importance of IL1β was shown also in a large clinical trial where a monoclonal antibody against IL1β reduced the rate of recurrent cardiovascular events in patients that sustained a prior myocardial infarction and showed residual inflammatory risk as assessed by C-reactive protein levels (7).

Neuroimmune guidance cues (NGCs) are key regulators of cell migration and positioning and have emerged as significant modifiers of inflammation and atherogenesis. NGCs consist of four protein families including Semaphorins, Netrins, Ephrins and Slits. While these guidance cues were originally found to be associated with the embryonic development of the nervous and vascular system (8), it is now clear they also play important regulatory roles in adult physiology (9, 10). In addition, genetic variants in genes related to the axonal guidance pathway are found to be enriched in CVD and several novel genetic risk loci for CVD contain NGC genes (11, 12). Indeed, dysregulation of NGCs, e.g., differential expression of Netrin-1 (NTN1), EphrinB2 (EFNB2) and Semaphorin3A (SEMA3A) in atheroprone regions of mouse aortic endothelial cells, have been shown to alter leukocyte adhesion and migration and thereby are proposed to contribute to atherosclerotic plaque formation (13). Likewise, multiple members of the semaphorin family of NGCs have been validated to play a role in atherosclerosis. Semaphorin7A (SEMA7A), Semaphorin3E (SEMA3E) and its receptor PlexinD1 (PLXND1) have been shown to be expressed in atherosclerotic lesions and mediate leukocyte trafficking (14, 15). In addition, increased serum levels of SEMA3E are associated with atherosclerosis in individuals with metabolic syndrome (16) and a polymorphism in the Semaphorin3F gene was found to significantly associate with myocardial infarction (17).

The semaphorin family comprises a large group of secreted, surface-attached or membrane-bound semaphorin ligands (SEMAs). Signaling of semaphorins is mainly mediated by the membrane-bound plexin receptors (PLXNs). Binding of semaphorins to plexin receptors, sometimes in combination with co-receptors, can induce signaling via the intracellular GTPase Activating Protein (GAP domain) and Rho GTPase Binding Domain (RBD domain) of the plexin receptors. As the activity and availability of small GTPases, which are key regulators in many cellular processes such as cytoskeletal dynamics, can be directly controlled by these domains, plexin signaling can hereby regulate for example cell morphology, cell migration and cell proliferation (18, 19). Besides having a crucial role in mammalian physiology, semaphorin-plexin signaling is involved in several pathophysiological processes like cancer, microvascular disease, osteoporosis and inflammatory diseases (20).

In this study we have shown that many NGCs, in particular several semaphorin family members, are differentially expressed in monocytes and endothelial cells by pro-inflammatory cytokines and that Plexin A4 (PLXNA4) may have a protective role in endothelial barrier function via modulation of the cellular cytoskeleton.

## Materials and Methods

### Database Expression Profiles

Expression of NGCs by monocytes and endothelial cells was determined using the GENEVESTIGATOR® software (21). All published data on the Affymetrix Human Genome U133 Plus 2.0 Array (HG0U133 Plus 2.0/GPL570) platform on NGCs expressed in human monocytes and endothelial cells was extracted and analyzed to select NGCs expressed.

### Primary Cells, Cell Lines and Media

#### Primary Monocytes

Peripheral blood mononuclear cells (PBMCs) were isolated from buffy coats from five individual healthy subjects (Sanquin, the Netherlands), obtained after informed written consent (Ethical Approval Number BTL 10.090). PBMCs were isolated by density gradient separation using Ficoll. CD14 Microbeads (Miltenyi Biotec, 130-050-201) and LS columns (Miltenyi Biotec, 130-042-401) were used for magnetic separation of CD14 positive monocytes. Isolated cells were maintained in RPMI 1640 medium (Gibco, 22409) supplemented with 10% FCS, 1% L-glutamine and 1% antibiotics (penicillin/streptomycin, Gibco, 15070063). For stimulation experiments monocytes were seeded at a density of 1.5 × 10^6^ cells/well in a 6-wells plate. Cells were stimulated with 20 ng/mL IL1β (PeproTech, #200-01B) or 10 ng/mL TNFα (Sigma, H8916) for 5 or 24 h. Unstimulated controls were taken along for both time points.

#### Endothelial Cells

Primary human umbilical vein endothelial cells (HUVECs) were isolated from human umbilical cords as described previously (22). Cells were cultured on gelatin (1%) coated surfaces. For initial expression experiments, endothelial cells were cultured in endothelial growth medium (EGM)-2 medium of Lonza (CC3156 supplemented with CC4176 and 1% antibiotics) while functional assays were performed in EGM2 medium of Promocell (C-22211 supplemented with C-39211 and 1% antibiotics). No differences were observed in culturing between the two kinds of medium. The human immortalized endothelial cell line ECRF (23) was cultured under similar conditions as primary endothelial cells. For stimulation experiments endothelial cells were seeded at a density of 0.5 x 10^6^ cells/well in a 6-wells plate. Cells were stimulated with 20 ng/mL IL1β or 10 ng/mL TNFα for 5 or 24 h. Unstimulated controls were taken along for both time points.

#### THP1 Cells

THP1 cells (ATCC®, TIB-202™) were cultured in RPMI 1640 medium supplemented with 10% FCS, 1% L-glutamine, 25 nM β-mercaptoethanol and 1% antibiotics.

### Quantitative PCR

Total RNA was isolated using TRIzol (Invitrogen, 15596018) and RNeasy Mini Kit (Qiagen, 74106) according to manufacturer's instructions. Total RNA was reverse transcribed using M-MLV Reverse Transcriptase Kit (Promega, M1701). qPCR analysis was conducted using SYBR Select Master Mix (Applied Biosystems, 4472908) and the forward and reverse primers as indicated in Supplementary Table 1. The PCR cycling conditions were: Initial denaturation at 95°C for 10 min, followed by 40 cycles at 95°C for 15 s, 60°C for 30 s and 72°C for 30 s, followed by a final extension step at 72°C for 10 min. mRNA expression was normalized to expression of GAPDH and represented as the natural logarithm of the fold change in expression compared to untreated or as copies per GAPDH.

### Lentiviral Transduction

To obtain a knockdown or overexpression of PLXNA4, endothelial cells were transduced with lentiviral particles. Knockdown of PLXNA4 was performed in HUVECs and was achieved by transduction with virus particles encoding a shRNA against the coding region of PLXNA4 (Mission library Sigma-Aldrich, TRCN0000078686). As a control, cells were transduced with lentiviral particles encoding a scrambled shRNA. Selection of transduced cells was achieved using puromycin (2 μg/mL).

A vector encoding the PLXNA4 gene was kindly provided by Prof. G. Neufeld. Due to low transduction efficiency in primary endothelial cells (caused by the large size of the PLXNA4 vector), overexpression of PLXNA4 was performed in the immortalized endothelial cell line ECRF. Transduced cells were selected using blasticidin (50 μg/mL). As a control, cells were transduced with a mock virus and selected with puromycin.

### Protein Analysis

Protein samples were collected from confluent 6-wells plates by cell lysis with Novex Tris-Glycine SDS sample buffer (ThermoFisher, LC2676) and subsequent sonication, or lysis with cold RIPA buffer (Cell signaling, 9806) and centrifugation at 14,000 rpm for 10 min at 4°C. Approximately 30–50 μg of protein sample was denatured using DTT at 90°C for 10 min. Proteins were size separated using 10% Mini-PROTEAN gels (Biorad, 4561033) and transferred to PVDF membranes (Biorad, 1704156) using the Trans-Blot Turbo system of Biorad. Membranes were blocked with 5% milk in TBST and incubated overnight with primary antibody against PLXNA4 (1:500, Novus, NBP1-85128), VE-cadherin (1:500, BD Biosciences, 555661), ICAM-1 (1:1000, Cell signaling, 4915), CDKN1A (1:500, Santa Cruz, SC-397) or GAPDH (1:1000, Cell signaling, 5174S). After incubation with Horseradish peroxidase (HRP-) conjugated secondary antibodies (1:5000, DAKO) membranes were developed with either Western lightning ECL (PerkingElmer, NEL1030001EA) or SuperSignal Westernblot Enhancer (ThermoFisher, 46640) and visualized with the ChemiDoc Touch Imaging System (Biorad). Pictures were analyzed using ImageLab software (Biorad) and expression was quantified using ImageJ software from the NIH.

### Immunofluorescence

Endothelial cells were seeded on ibiTreat 8-well μ-Slides (Ibidi, 80826) at a density of 30.000 cells/well. After a 10-min fixation in 4% paraformaldehyde in Hank's Balanced Salt Solution supplemented with calcium and magnesium (HBSS++, Gibco, 14025092), cells were permeabilized with 0.1% TritonX-100 in HBSS++ for 2 min. Non-specific antigens were blocked with 5% BSA in HBSS++ followed by incubation with primary antibody against VE-Cadherin (3.33 μg/mL, Becton Dickinson, 555661) at room temperature for 1 h, or CDKN1A (2 μg/mL, Santa Cruz Biotechnology, SC-397-G) at 4°C overnight. After incubation with Hoechst (5 μg/mL, Molecular Probes, H-3569), rhodamine-conjugated phalloidin (0.5 μg/mL, Sigma-Aldrich, P1951) and appropriate secondary antibody, donkey-anti-mouse Alexa488 (2 μg/mL, Molecular Probes, A21202) or donkey-anti-goat Alexa 488 (4 μg/mL, Molecular Probe, A11055) at room temperature for 1 h, excess staining was washed off and cells were covered with HBSS++. Overview pictures were taken with the TCS SP5 confocal microscope (Leica) or SP8 Confocal WLL microscope (Leica) and quantification was performed. Relative area and intensity were determined by adaptive thresholding using either ImageJ or R-studio (version 1.3.959), and the EBImage package (version 4.29.2) (24). VE-cadherin and F-Actin total fluorescence is represented as fold change of (positive area^*^mean intensity)/number of nuclei compared to control cells. For quantification of the distribution of VE-cadherin and F-actin (border/interior), each image was segmented into individual cells. Resulting segmentations were used to determine the cell borders and cell interiors from which total immunofluorescent signal intensities were extracted. Signal intensities were corrected for total area of that segment and the ratio of border intensity vs. interior intensity was plotted. For CDKN1A quantification Hoechst positive nuclei were selected and mean fluorescence intensity of CDKN1A was measured.

### Proliferation Assay

Cells were seeded at a density of 20.000 cells/well in 12-wells plates in triplicate. On day 0, 1 or 3 after seeding cells were incubated with 500 μg/mL Methylthiazolyldiphenyl-tetrazolium bromide (MTT) in PBS for 30 min at 37°C. Subsequently, MTT suspension was removed and cells were lysed using isopropanol/0.04 M HCl. Lysates were transferred to a 96-wells plate and absorbance at 562 nm was measured using the Spectramax M2 plate reader. Proliferation rates are expressed as fold change in absorbance compared to baseline measurement at day 0.

### Migration Assay

Endothelial cells were seeded at a density of 21.000 cells/well into 2 well-culture inserts (Ibidi, 80209) placed in 24-wells plates in triplicate. After the cells reached confluency, inserts were removed and cells were placed on low serum medium (EBM2 with 0.5% FCS and 1% Pen/Strep) containing 100 nM phorbol 12-myristate 13-acetate (PMA) to activate endothelial cells to migrate (25, 26). At different time intervals (0, 2, 4, 6, 8, and 24 h), images were taken and gap closure was assessed using ImageJ. Wound closure is expressed as percentage open area compared to baseline at time = 0 set at 100%.

### Barrier Function (TEER)

Endothelial barrier function was assessed by measuring trans-endothelial electrical resistance (TEER) using the electric cell-substrate impedance sensing system (ECIS Zθ, Applied Biophysics). ECIS plates (96W20idf PET, Applied Biophysics) were pretreated with L-Cystein and coated with 1% gelatin. After taking baseline measurements, endothelial cells with and without knockdown or overexpression were added to the plate in the absence or presence of 10 μM Y-27632 ROCK inhibitor (StemCell technologies, 72302). Multiple frequency/time (MFT) mode was used for real-time assessment of the barrier. Results are expressed as relative resistance at frequency of 4,000 Hz corrected for baseline resistance. ECIS software was used for further mathematical modeling. Using impedance data, this model enables to calculate the cell morphological parameters cell-cell (Rb) and cell-matrix (α) contacts (27, 28).

In addition, stable endothelial barriers were modulated by addition of 10 μM Y-27632 ROCK inhibitor or 1 μg/mL recombinant SEMA3A (R&D Systems, 1250-S3). Results are expressed as percentage of the average barrier of the endothelial cells measured over 4–5 h before addition of stimuli.

### Tube Formation Assay

Cells were seeded at a density of 15.000 cells/ml in an angiogenesis μ-slide (Ibidi, 81506) on top of solidified Matrigel (Growth factor reduced, Corning, 354230). Using the live cell imaging system ImageXpress (Molecular Devices), images were acquired over time. From the images, the capillary forming capacity of endothelial cells was quantified using the Angiogenesis Analyzer (http://image.bio.methods.free.fr/ImageJ/) in imageJ.

### Permeability Assay

Endothelial cells were cultured as 3D capillary-like vessels using the Organoplate® microfluidic system (Mimetas, 9603-400B) and used for permeability assays based on the method described by van Duinen et al. (29). First, a collagen-1 gel was patterned in the gel channel using 2 μL of a neutralized collagen-1 solution as described before. The plates were incubated for 10 min at 37°C to establish polymerization of the collagen-1 gel. Before cell seeding, perfusion channels were filled with 25 μL of 1% gelatin and incubated for 30 min at 37°C to enable coating of the perfusion channel. To prevent dehydration, 25 μL of HBSS++ was added on top of the gel inlet. After incubation, the gelatin was replaced with 50 μL of EGM2 medium and cells were seeded using the passive pumping method (30) by adding 1 μL of endothelial cell suspension at a concentration of 2 x 10^7^ cells/mL, to the perfusion outlet. The plates were incubated in a static way for 1 h at 37°C to allow for adherence of the cells to the coated surface. Hereafter, another 50 μL of EGM2 medium was added to the perfusion outlet. The plate was placed in an upright position on an interval rocker platform with a 7° inclination and 8 min cycle time, to allow for continuous perfusion of the capillary-like vessels. Medium was refreshed three times a week and after 7 days the capillary-like vessels were used to assess permeability. Alexa 555-labeled albumin (75 μg/mL, Invitrogen, A34786) was added to the perfusion channel and leakage of albumin to the gel channel over a time period of 30 min was assessed with the ImageXpress confocal microscope (Molecular Devices). Quantification was performed as described before (29) using ImageJ. In short, fluorescent intensities were quantified in selected regions of interest and apparent permeability [P_app_ (cm/s)] was calculated using the following formula;

$$\begin{array}{r} P_{app}=\frac{d\left(\frac{I_{g}}{I_{p}}\right)}{dt}•\frac{A_{g}}{l_{w}} \end{array}$$

where I_g_ is the intensity in the gel channel, I_p_ the intensity in the perfusion channel, t is time in seconds, A_g_ the surface area of the capillary-like vessel that is in contact with the gel (cm^2^) and l_w_ the length of the phaseguide (cm).

### Trans-Endothelial Migration

Chemotaxis of THP1 monocytes through a monolayer of endothelial cells was measured using 24-well Boyden chamber with a 5 μm pore size filter (Corning, 734-1573). Endothelial cells were seeded on top of the filter at a density of 50,000 cells/well and incubated for 2–3 days to form a stable monolayer after which monocyte migration across the endothelial monolayer toward 10 ng/mL recombinant human monocyte chemotactic protein (MCP-1, R&D Systems, 279-MC) was measured. After 3 h, cells in the lower chamber were resuspended, microscopic pictures were taken and the number of migrated THP1 cells was determined with cell count. Each condition was performed in triplicate and migration is expressed relative to migration of control cells toward MCP-1, set at 1.

### Monocyte-Endothelial Adhesion

THP1 cells were labeled with 5 μg/mL Calcein AM (Molecular Probes Life Technologies, C3100MP) and incubated on top of a monolayer of endothelial cells for 30 min at 37°C. Non-adhering cells were washed away by multiple washing steps with PBS after which the remaining adhered cells were lysed in 0.5% TritonX-100 for 10 min. Fluorescence was measured at λex 485 nm and λem 514 nm with the Spectramax M2 plate reader (Molecular Devices). Each condition was performed in quadruplo. Monocyte adhesion is represented as fold change in fluorescence compared to control cells, set at 1.

### RAC-1 Activity Assay

RAC-1 activity of endothelial cells with normal or decreased expression of PLXNA4 was assessed by a CRIB-peptide based pull-down assay (31). In short cells were lysed in lysis buffer (150 mM NaCl, 50 mM Tris pH 7.6, 1% Triton X-100, 20 mM MgCl_2_) with protease inhibitors. After centrifugation, lysates were incubated with 50 ng of CRIB peptide (32) and streptavidin agarose beads for 30 min rotating at 4°C. Accordingly, bound RAC-1 was detected by western blotting with RAC-1 antibody (1:500, BD Bioscience, 610651) as described earlier.

### RhoA Activity G-Lisa

Serum-starved endothelial cells with or without PLXNA4 knockdown were collected and processed for G-LISA RhoA activation assay (Cytoskeleton, BK124) according to manufacturers' instruction. Results are expressed as fold change in absorbance relative to control cells.

### Statistical Analyses

Differences between two groups were analyzed with two-tailed unpaired *t*-tests. For groups with unequal variances Welch correction was applied. Two-way repeated measures ANOVA tests followed by Sidaks multiple comparison tests were used to test the difference between multiple groups over time. Two-sided *P*-values of < 0.05 were considered statistically significant. All statistical analysis were performed with SPSS version 24 or Graphpad Prism 8.

## Results

### Neuroimmune Guidance Cues Are Differentially Regulated by Pro-Inflammatory Stimuli

Combining publicly available gene expression data, the highest expressed NGCs in monocytes (Supplementary Table 2) and endothelial cells (Supplementary Table 3) were selected for determination of its expression under pro-inflammatory conditions. Freshly isolated monocytes and endothelial cells from healthy individuals were stimulated with IL1β or TNFα for 5 or 24 h. Using quantitative PCR, changes in expression of the selected genes upon stimulation with IL1β or TNFα were analyzed.

Stimulation of monocytes with IL1β or TNFα for 5 h significantly increased expression of *PLXNC1* and *SEMA3C* compared to unstimulated cells (Supplementary Figure 1A), which both were no longer increased after 24 h of stimulation (Supplementary Figure 1B). However, stimulation of monocytes for 24 h with IL1β and/or TNFα did result in significant downregulation of the genes *EPHB6, EFNA4, EFNB1, NRP2, PLXNC1, PLXND1, SEMA3F, SEMA4A, SEMA4D, SEMA6B*, and *SEMA6C* (Supplementary Figure 1B).

**Figure 1:**
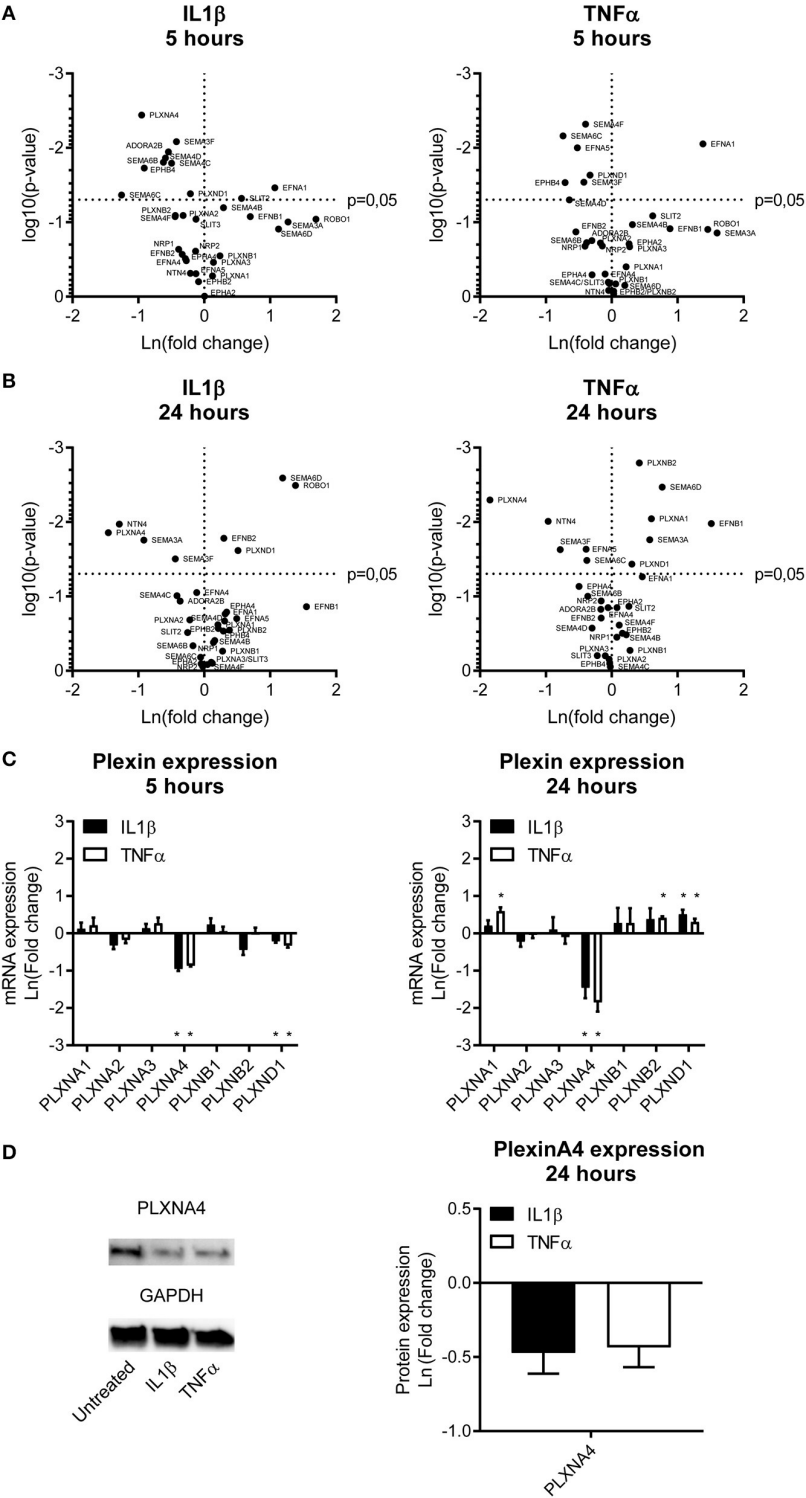
Endothelial expression of NGCs under pro-atherogenic conditions. **(A,B)** Volcano plots depicting up and down regulation of NGCs after stimulation of primary human endothelial cells with IL1β (20 ng/mL) or TNFα (10 ng/mL) for **(A)** 5 or **(B)** 24 h. Results are depicted as mean of the natural logarithm transformed fold change in expression compared to unstimulated cells and plotted against significance. *N* = 4. **(C)** Quantitative PCR of mRNA expression of plexin receptors in endothelial cells after 5 h or 24 h of stimulation with IL1β (20 ng/mL) or TNFα (10 ng/mL). Data is depicted as mean of the natural logarithm transformed fold change in expression compared to unstimulated cells. Mean ± S.E.M. of *N* = 4, *P < 0.05. **(D)** Immunoblots and quantification of PLXNA4 and GAPDH protein in primary human endothelial cells stimulated with IL1β (20 ng/mL) or TNFα (10 ng/mL) for 24 h. Results are depicted as fold change in intensity. Mean ± S.E.M. of *N* = 4.

Stimulation of endothelial cells with the same pro-inflammatory cytokines resulted in a different expression pattern compared to monocytes. A 5h stimulation with either IL1β or TNFα downregulated gene expression of *EPHB4, EFNA5, ADORA2B, PLXNA4, PLXND1, SEMA3F, SEMA4C, SEMA4D, SEMA4F, SEMA6B*, and *SEMA6C*. Gene expression of *EFNA1* and *SLIT2* were significantly upregulated (Figure 1A). After 24 h of incubation with IL1β or TNFα more genes showed increased expression, namely *EFNB1, EFNB2, ROBO1, PLXNA1, PLXNB2, PLXND1, SEMA3A*, and *SEMA6D*. Expression of *EFNA1* remained slightly increased while expression of *SLIT2* returned to normal. Downregulation of *EFNA5, PLXNA4, SEMA3F*, and *SEMA6C* gene expression was still observed after 24 h and additionally *NTN4* expression was significantly reduced after 24h stimulation. Expression of *SEMA3A*, which is only moderately expressed in endothelial cells, also showed significant changes after 24 h of endothelial stimulation, but was decreased upon IL1β and increased upon TNFα stimulation (Figure 1B).

### Pro-Inflammatory Cytokines Decrease the Expression of PLXNA4 in Endothelial Cells

A differential expression of several NGC ligands and receptors was observed in endothelial cells and monocytes subjected to pro-inflammatory stimuli, with most significant changes in semaphorin ligands and its plexin receptors. Therefore, the plexin receptors in endothelial cells were further examined (Figure 1C). Expression of *PLXNA4* and *PLXND1* was affected most by inflammation. While 5 h of stimulation with IL1β and TNFα resulted in a decreased expression of *PLXND1*, after 24 h of stimulation *PLXND1* expression was increased (Figure 1C). A more pronounced downregulation after 5 h of stimulation with both IL1β and TNFα was observed for *PLXNA4*. The 60% reduction in expression after 5 h was even stronger, up to ~ 80%, after 24 h. In addition to a decrease in mRNA expression, a decrease in PLXNA4 protein expression was detected after 24 h of exposure of endothelial cells to IL1β and TNFα (Figure 1D).

### Downregulation of PLXNA4 in Endothelial Cells Alters the Cellular Phenotype

**Figure 2:**
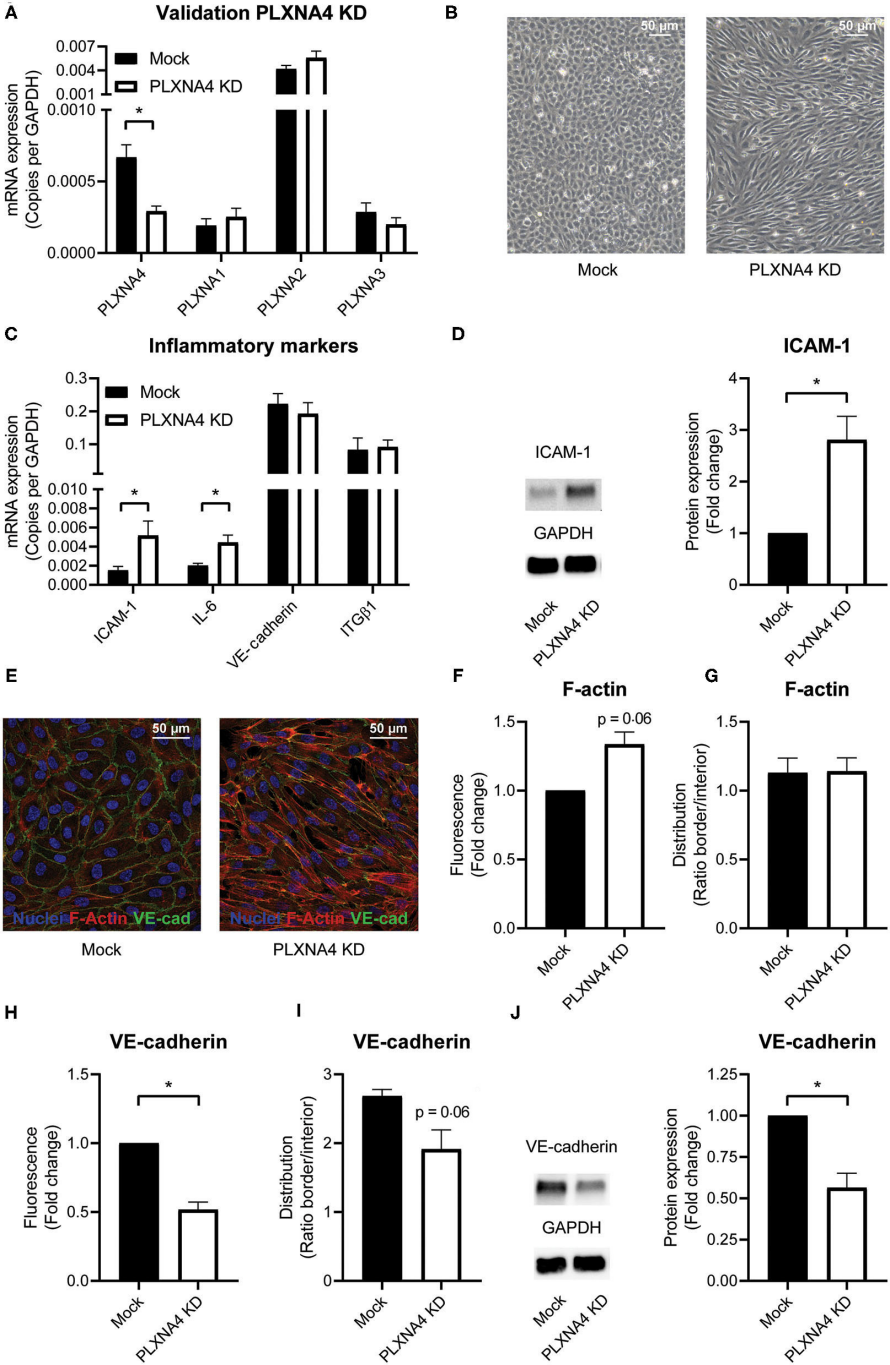
PLXNA4 downregulation alters endothelial cellular phenotype. **(A)** mRNA expression of PLXNA4, PLXNA1, PLXNA2, and PLXNA3 in endothelial cells treated with a lentiviral non-targeting shRNA (mock) or a shRNA against PLXNA4 (PLXNA4 KD). Results are expressed as copies corrected for GAPDH. Mean ± S.E.M. of *N* = 27, *P < 0.05. **(B)** Representative overview pictures of mock-treated and PLXNA4 KD endothelial cells. **(C)** mRNA expression of ICAM-1, IL-6, VE-cadherin and integrinβ1 (ITGB1) in mock or PLXNA4 KD endothelial cells. Results are expressed as copies corrected for GAPDH. Mean ± S.E.M. of *N* > 4, *P < 0.05. **(D)** Immunoblots and quantification of ICAM-1 and GAPDH protein in mock control cells and PLXNA4 KD cells. Results are depicted as fold change in intensity. Mean ± S.E.M. of *N* = 7. **(E–I)** Immunofluorescent staining of F-actin (red), VE-cadherin (green) and nuclei (blue) in mock or PLXNA4 KD endothelial cells. **(E)** Representative fluorescent overview photographs. **(F–I)** Quantification of **(F)** F-actin fluorescent signal, **(G)** F-actin cellular distribution, **(H)** VE-cadherin fluorescent signal or **(I)** VE-cadherin cellular distribution. Fluorescent signal is quantified as (mean fluorescent intensity*area)/nuclei and expressed as fold change relative to control cells. The cellular distribution is quantified as mean intensity per pixel of the border or interior area and expressed as the ratio between border and interior. Mean ± S.E.M. of *N* = 3, *P < 0.05. **(J)** Immunoblots and quantification of VE-cadherin and GAPDH protein in control cells and PLXNA4 knockdown cells. Results are depicted as fold change in intensity. Mean ± S.E.M. of *N* = 4.

To understand the functional consequences of changes in expression of PLXNA4 by endothelial cells, endothelial cells with either shRNA-mediated knockdown of PLXNA4 (PLXNA4 KD) or overexpression of PLXNA4 (PLXNA4 OE) were generated. Upon knockdown, an approximately 60% reduction in mRNA expression compared to mock-treated control cells (Mock) was achieved while expression of other PLXNA receptors was unaffected (Figure 2A). Stable overexpression of PLXNA4 in the endothelial cell line ECRF resulted in a 70-fold increase of PLXNA4 mRNA (Supplementary Figure 2A). Reduced levels of PLXNA4 led to a striking alteration in endothelial morphology giving them a more elongated shape and inflammatory-like phenotype (Figure 2B). Indeed, expression of the inflammatory markers IL-6 and ICAM-1 were increased in endothelial cells with decreased expression of PLXNA4 (Figures 2C,D). Characterization of the F-actin cytoskeleton revealed an increased overall signal of F-actin in the PLXNA4 knockdown cells compared to mock-treated control cells (Figures 2E,F), while the distribution of F-actin over the cell border and the interior of the cell was comparable (Figure 2G). In addition, VE-cadherin cell-cell junctions were highly altered in the PLXNA4 knockdown cells compared to the mock-treated control cells (Figures 2E,H,I). Not only was the overall signal for VE-cadherin lower in PLXNA4 KD endothelial cells (Figure 2H), we also observed less incorporation of VE-cadherin into the cellular border (Figure 2I). The decreased expression of VE-cadherin protein in PLXNA4 knockdown endothelial cells was validated with Western blot protein analysis (Figure 2J). In contrast to all observed changes in PLXNA4 knockdown cells, overexpression of PLXNA4 in endothelial cells did not lead to differences in morphology and cell-cell interactions (Supplementary Figures 2B–E).

### Loss of Endothelial PLXNA4 Decreases Cell Proliferation and Impairs Endothelial Barrier Formation and Function

**Figure 3:**
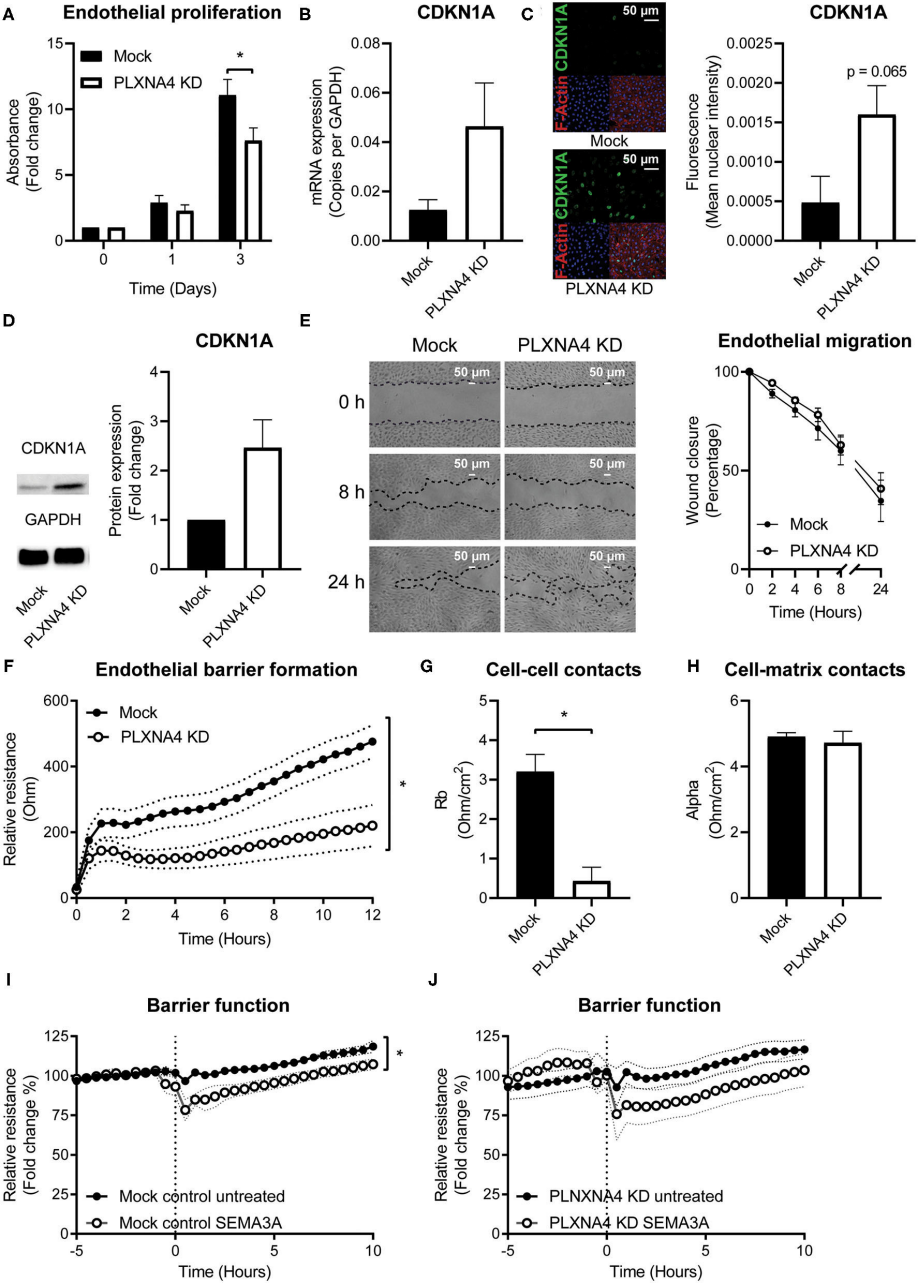
Loss of endothelial PLXNA4 reduces cell proliferation and barrier function. **(A)** Proliferation of mock and PLXNA4 KD endothelial cells. Results are expressed as fold change to day 0 set at 1. Mean ± S.E.M. of *N* = 6, *P < 0.05. **(B)** mRNA expression of CDKN1A in mock or PLXNA4 KD endothelial cells. Results are expressed as copies corrected for GAPDH. Mean ± S.E.M. of *N* = 8. **(C)** Representative overview photographs and quantification of CDKN1A staining of mock and PLXNA4 KD endothelial cells. Results are expressed as fluorescent intensity in the nuclei of the cells. Mean ± S.E.M. of *N* = 4. **(D)** Immunoblots and quantification of CDKN1A and GAPDH protein in control cells and PLXNA4 knockdown cells. Results are depicted as fold change in intensity. Mean ± S.E.M. of *N* = 4. **(E)** Representative overview photographs and quantification of migration of mock and PLXNA4 KD endothelial cells over time. Results are presented as percentage of open area. Mean ± S.E.M. of *N* = 6. **(F)** Trans-endothelial electrical resistance of mock and PLXNA4 KD endothelial cells over time. Mean ± S.E.M. of *N* = 5, *P < 0.05. **(G,H)** Endothelial electrical resistance attributable to **(G)** cell-cell contacts (Rb) and **(H)** cell-matrix contacts (alpha) in a monolayer of mock and PLXNA4 KD endothelial cells. Mean ± S.E.M. of *N* = 5, *P < 0.05. **(I, J)** Effect of addition of 1 μg/mL SEMA3A to a stable monolayer of **(I)** mock-treated control or **(J)** PLXNA4 KD endothelial cells, on endothelial barrier function. Relative resistance is expressed as percentage of the average barrier before stimulation. Mean ± S.E.M. of *N* = 5, *P < 0.05.

As endothelial cells are key regulators of vascular homeostasis, we set out to investigate the effect of PLXNA4 on endothelial proliferation, migration and barrier function. Using a MTT-based proliferation assay, the effect of PLXNA4 expression on proliferation rates of endothelial cells was determined. Proliferation rates in PLXNA4 knockdown cells were significantly lower after 3 days compared to control cells (Figure 3A). In line with this finding, the expression of CDKN1A, an inhibitor of cell cycle progression, is increased in endothelial cells with decreased expression of PLXNA4 at both mRNA and protein level (Figures 3B–D). Endothelial cells overexpressing PLXNA4 showed no difference in proliferation compared to control cells (Supplementary Figure 3A). Migratory capacity in the presence of PMA was not affected by reduced nor increased levels of PLXNA4 (Figure 3E and Supplementary Figures 3B,C). Next, the endothelial barrier function was assessed by TEER measurements of endothelial cells. Reduction of PLXNA4 expression decreased the capacity of endothelial cells to form a tight barrier (Figure 3F). Applying further mathematical modeling using the provided ECIS software, revealed that the decreased barrier upon reduction of PLXNA4 was primarily caused by less efficient cell-cell contacts (Figure 3G), while cell-matrix contacts were not affected (Figure 3H). Activation of endothelial cells with the cognate ligand for the PLXNA4 receptor, SEMA3A, significantly decreased barrier function in mock-treated control cells (Figure 3I). However, a decreased barrier function upon SEMA3A addition, although not significant, was also observed for PLXNA4 knockdown cells (Figure 3J).

Consistent with the unchanged morphology, VE-cadherin junction localization and actin fibers, TEER measurements of endothelial cells with PLXNA4 overexpression showed no difference in barrier function compared to their control endothelial cells (Supplementary Figures 3D–F).

### Loss of Endothelial PLXNA4 Diminishes Endothelial Tube Formation and Induces Vascular Leakage

**Figure 4:**
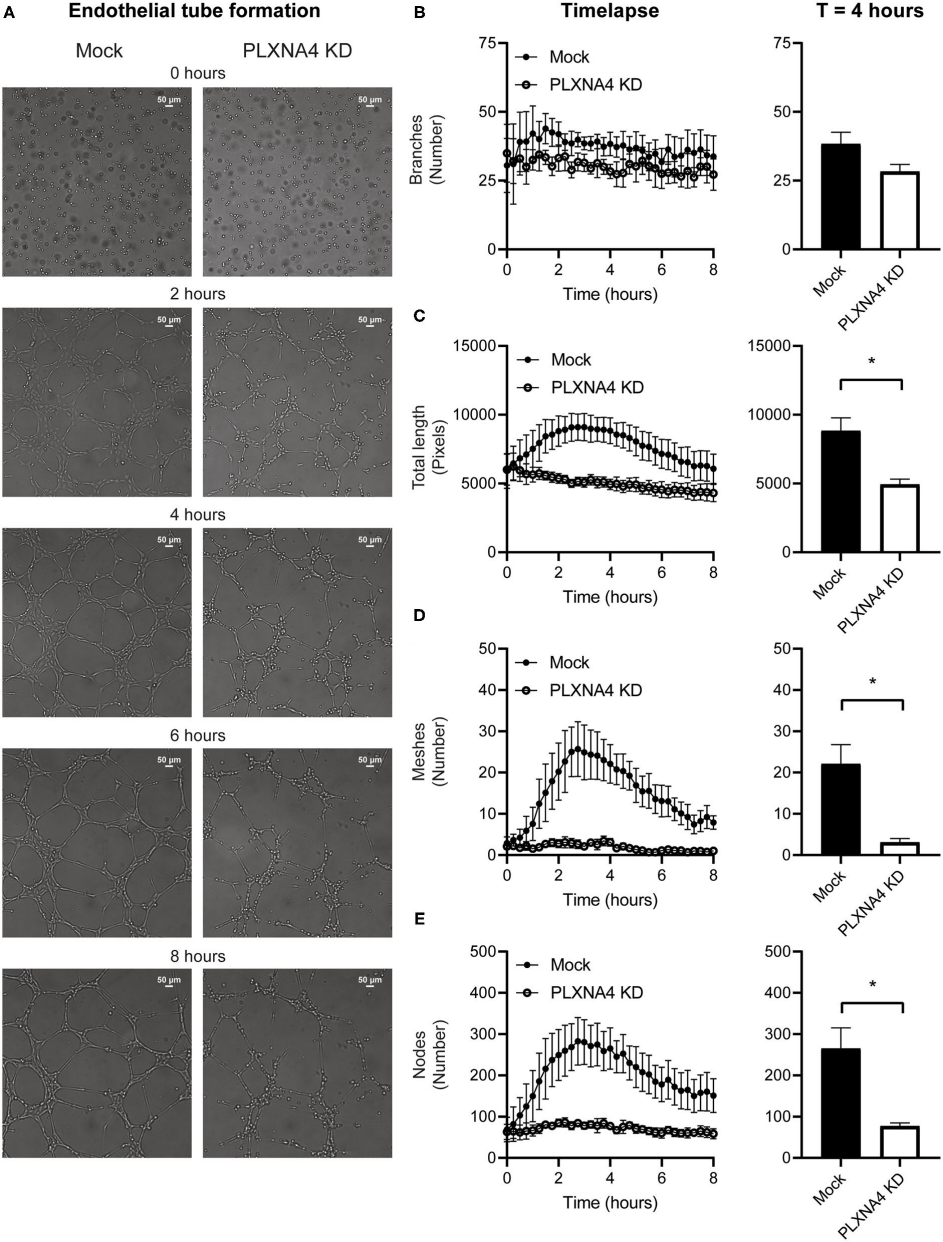
Loss of endothelial PLXNA4 reduces tube-like structure formation. **(A)** Representative overview photographs and **(B–E)** quantification of tube formation of mock and PLXNA4 KD endothelial cells over time. Results are presented as **(B)** number of branches, **(C)** total length in pixels, **(D)** number of meshes and **(E)** number of nodes over time (left graph) and at 4 h (right graph). Mean ± S.E.M. of *N* = 3, *P < 0.05.

As a decrease in endothelial cell-cell contacts upon knockdown of PLXNA4 was observed the capacity of endothelial cells to form tube-like structures, another important property of endothelial cells, was assessed. A Matrigel-based tube formation assay showed a decreased ability of endothelial cells with reduced levels of PLXNA4 to form tube-like structures (Figure 4A). While the amount of initial branches (Figure 4B) did not differ between conditions, endothelial cells with less PLXNA4 form significantly less tube-like structures, quantified as diminished total tubule length (Figure 4C), number of meshes (Figure 4D) and number of nodes (Figure 4E) after 4 h of capillary formation.

**Figure 5:**
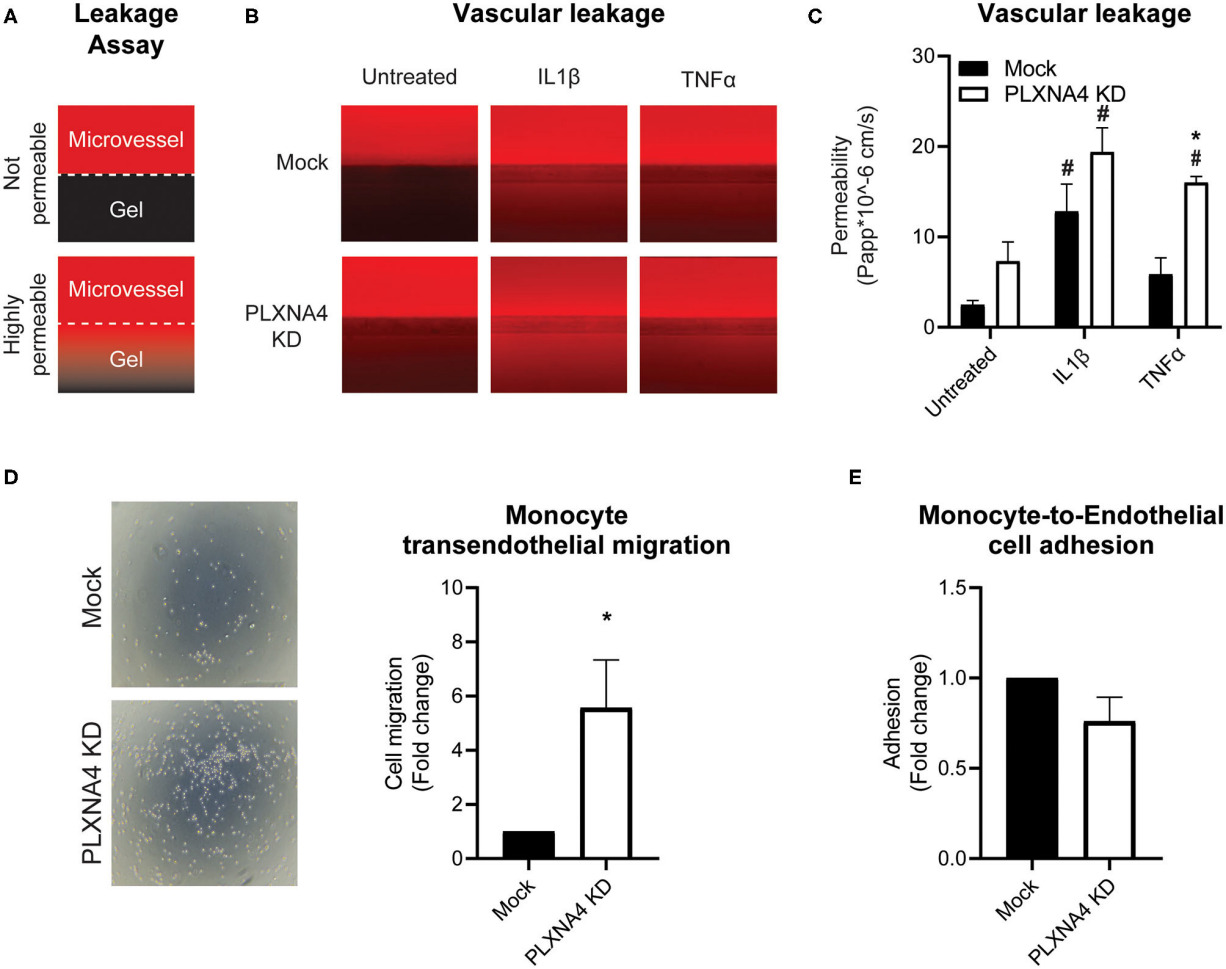
Loss of endothelial PLXNA4 induces vascular permeability. **(A)** Schematic overview of the leakage assay principle, where 3D cultured capillary-like vessels in the upper perfusion channel are separated from the collagen gel in the lower channel with a phaseguide. Leak tight capillary-like vessels will have no to limited leakage of red fluorescent-labeled albumin from the perfusion channel to the gel, while increased permeability of capillary-like vessels will result in increased fluorescent signal in the gel channel. **(B)** Representative overview photographs and **(C)** quantification of the apparent permeability of capillary-like vessels composed of endothelial cells with normal or decreased expression of PLXNA4 and with or without IL1β (20 ng/mL) or TNFα (10 ng/mL) stimulation. Mean ± S.E.M. of *N* = 4, *P < 0.05 for mock transduced cells vs. PLXNA4 KD cells ^#^*P* < 0.05 for stimulated cells vs. untreated cells. **(D)** Representative overview pictures and quantification of migrated monocytes through a monolayer of mock or PLXNA4 KD endothelial cells toward MCP-1. Results are relative to mock control cells, set at 1. Mean ± S.E.M. of *N* = 8. **(E)** Quantification of adhered fluorescently labeled monocytes to mock and PLXNA4 KD endothelial cells. Results are relative to mock control cells, set at 1. Mean ± S.E.M. of *N* = 9.

Next, the ability of PLXNA4 knockdown endothelial cells to form leak tight 3D capillary-like vessels was assessed using the Organoplate® microfluidic system. Perfusion of the capillary-like vessels with fluorescently labeled albumin and measuring its leakage into the neighboring gel channel (Figure 5A), indicated an increased leakage in capillary-like vessels composed of PLXNA4 knockdown endothelial cells compared to control capillary-like vessels. Exposure of capillary-like vessels to IL1β significantly induced vascular leakage in both control and PLXNA4 knockdown cells, while TNFα only significantly induced leakage in PLXNA4 knockdown cells (Figures 5B,C).

In line with increased permeability to solutes, endothelial cells with decreased expression of PLXNA4 KD cells were also more permeable to immune cells. Using a Transwell filter covered with control or PLXNA4 knockdown endothelial cells it was shown that monocyte migration toward MCP-1 was increased when a monolayer of endothelial cells with decreased PLXNA4 expression was present (Figure 5D), while adhesion was not affected (Figure 5E).

### Loss of Endothelial PLXNA4 Affects RhoA/ROCK Pathway Activity

**Figure 6:**
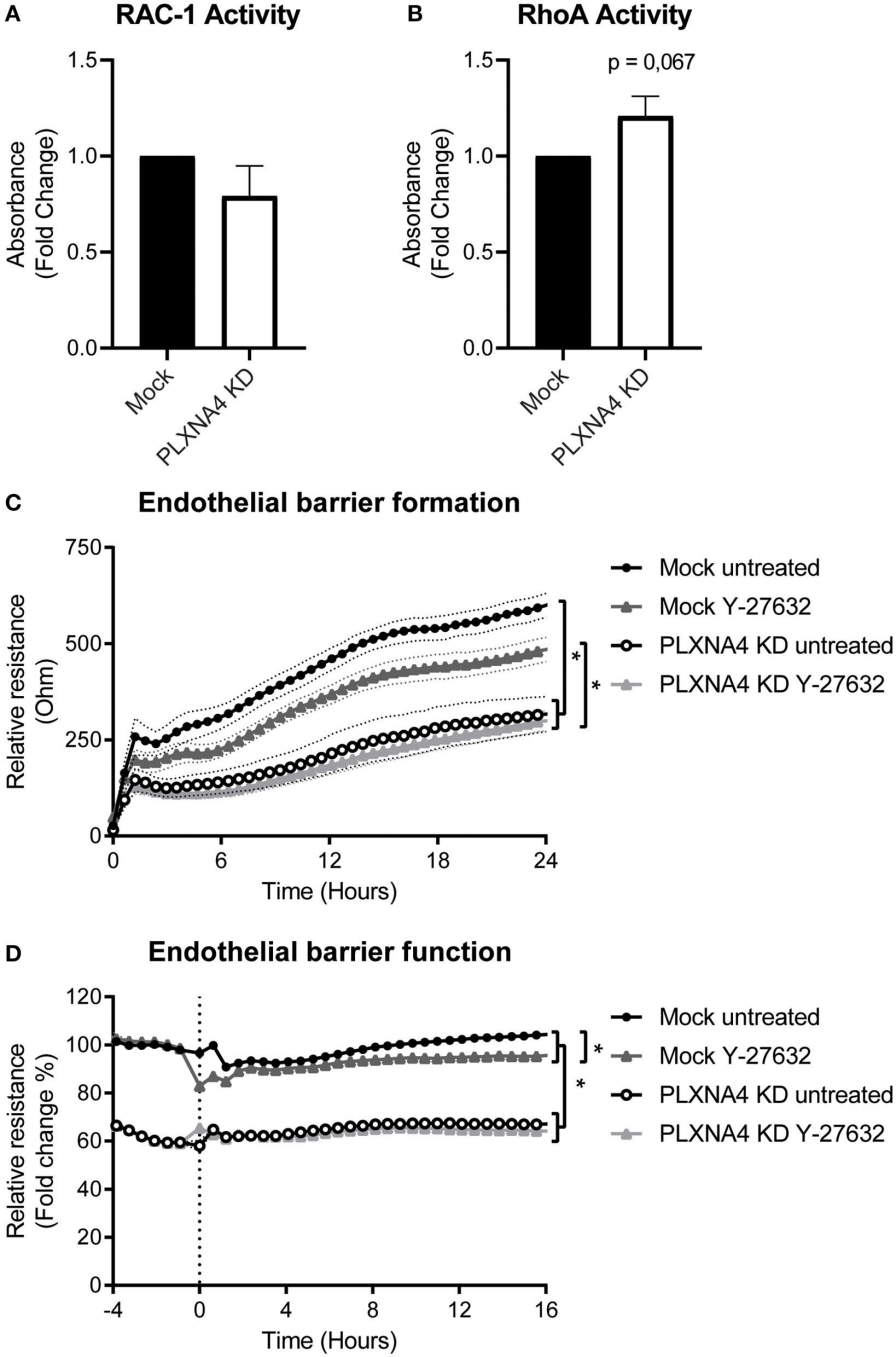
Loss of endothelial PLXNA4 affects RhoA/ROCK pathway activity. **(A,B)** Activity of the small GTPases **(A)** RAC-1 or **(B)** RhoA in mock-treated or PLXNA4 KD endothelial cells. Results are expressed as fold change in absorbance compared to control cells, set at 1. Mean ± S.E.M. of *N* = 8, *P < 0.05. **(C,D)** Effect of 10 μM Y-27632 ROCK inhibitor on **(C)** barrier formation and **(D)** barrier function of mock control cells or PLXNA4 knockdown cells. Results are expressed as or as percentage of the average resistance of untreated control cells, respectively. Mean resistance ± S.E.M. of *N* = 3, *P < 0.05.

Small GTPases are key regulators of many cellular processes. To investigate a potential mechanisms for the effect of PLXNA4 knockdown on endothelial function, the activity of small GTPases RAC-1 and RhoA were determined. While no clear difference in RAC-1 activity was observed (Figure 6A), an small increase in RhoA activity was detected in PLXNA4 knockdown cells compared to mock-treated control cells (Figure 6B). To further investigate the potential effect of RhoA on the increased vascular permeability of PLXNA4 knockdown cells, TEER experiments were performed with the addition of the ROCK inhibitor Y-27632. Exposure of endothelial cells to the Y-27632 ROCK inhibitor decreased the barrier formation capacity of mock-treated control cells (Figure 6C), but not as strong as PLXNA4 knockdown does. While PLXNA4 knockdown cells had an overall stronger impaired barrier formation capacity, no effect was observed upon treatment with the ROCK inhibitor. In addition, treatment of a stable monolayer of endothelial cells with the ROCK inhibitor resulted also in a significant decrease of the endothelial barrier of mock control cells, which was again not observed for endothelial cells with decreased expression of PLXNA4 (Figure 6D).

## Discussion

This study provides an extensive overview of the regulation of expression of a large number of NGC genes in atherosclerosis-related cell types under pro-inflammatory conditions. We have shown differential expression of NGC ligands and receptors with several changes in the Eph family of ephrin ligands and Eph receptors, but with most striking changes in the Semaphorin family with its semaphorin ligands and plexin receptors. The observed differential expression of several NGCs in this study strengthens the association between inflammation and NGCs and their potential effect on atherogenesis.

Moreover, this study demonstrates an important role in maintaining vascular integrity for PLXNA4 in endothelial cells, which is consistently downregulated with inflammation. Plexins are expressed in a variety of cells and have been described to regulate not only the developing and mature nervous and vascular system but also to modulate immune responses (20, 33). For example, both PLXNA1 (34) and PLXND1 (35) are involved in regulating T-cell activation and blockage of PLXND1 abolishes macrophage migration toward SEMA4A (36). In addition, dendritic cell migration is modulated by PLXNB1/SEMA4D signaling (37) and the motility of macrophages is affected by PLXNB2 (38). PLXNA4 has been shown to regulate T cells as PLXNA4-deficient mice have heightened T cell responses without altering lymphocyte development (39). In contrast to this immunosuppressive effect, PLXNA4 is also responsible for TLR-induced inflammatory cytokine production and PLXNA4-deficient mice are protected from the septic inflammatory response in a peritonitis model (40). While being expressed in endothelial cells as well, little is has been written about the role of PLXNA4 in adult endothelial cells (9), especially in an inflammatory setting. Here, we have shown that a decrease of PLXNA4 in endothelial cells resulted in cells with a stressed inflammatory and more elongated morphology and less cell-cell contacts when cultured in monolayers. In contrast, a study by Kigel and colleagues showed a more rounded morphology upon knockdown PLXNA4, but these endothelial cells were cultured as single cells (41). Functional assays, however, similarly showed a decrease in proliferation and tube formation in cells with decreased expression of PLXNA4.

In addition, our data showed an important role for PLXNA4 in maintaining vascular integrity via regulation of cellular morphology and maintaining the endothelial barrier. While several semaphorin ligands and/or plexin receptors have been described to play a role in regulating epithelial and endothelial barriers (42), the role of PLXNA4 herein has, to the best of our knowledge, not been described before. We have shown that endothelial cells with decreased expression of PLXNA4 also have decreased protein expression of the cell-cell contact protein, VE-cadherin. Moreover, incorporation of this protein the cellular border is hampered, overall resulting in a decreased barrier function of endothelial cells with decreased expression of PLXNA4. In accordance with the decrease in barrier function is our finding of increased vascular leakage of both solutes and monocytic cells *in vitro*. As vascular leakage is an important determinant in atherogenesis (43), increased vascular leakage as a result of decreased PLXNA4 expression suggests that PLXNA4 could be a determinant in atherogenesis.

While a decrease of PLXNA4 induced alterations in endothelial morphology, proliferation and barrier function, overexpression of PLXNA4 did not result in any significant alterations in morphology or function of endothelial cells. This suggests that a certain level of PLXNA4 is necessary for normal endothelial functioning while increased availability does not further protect endothelial cells against endothelial dysfunction. However, it should be noted that PLXNA4 overexpressing cells were generated from immortalized endothelial cells instead of primary endothelial cells as transduction efficiency of the large PLXNA4 vector was insufficient to transduce primary endothelial cells. As could be noticed with our morphology and immunofluorescent pictures, immortalized cells appear somewhat different from primary endothelial cells, which impairs the comparison between our PLXNA4 overexpression and knockdown model. Future experiments with more suitable cell models are necessary to confirm our hypothesis that a certain level of PLXNA4 is necessary for normal endothelial functioning.

Restoration of PLXNA4 expression levels is an interesting target to improve or even restore endothelial function and prevent progression of atherosclerosis. To enable cell-specific targeting of PLXNA4, more in-depth characterization of the regulation of PLXNA4 expression in endothelial cells, at both transcriptional and translational level, as well as PLXNA4's upstream and downstream targets is necessary. Plexin receptors have several signaling domains including domains that regulate the activity of protein kinases and small GTPases (44). Small GTPases, especially the Rho family of GTPases, are known for their regulation of cytoskeletal structures and can, amongst others, regulate cell division, migration and contraction (45) and therewith biological processes such as vascular integrity. While RhoA is described to be both barrier-disruptive and -protective, RAC-1 and Cdc42 are thought to be primarily involved in maintaining barrier integrity (46). We have shown that knockdown of PLXNA4 in endothelial cells had no effect on RAC-1 activity but increased RhoA activity, suggesting a potential barrier-disruptive effect of RhoA in these cells. TEER experiments in the presence and absence of a ROCK inhibitor revealed that barrier formation and function of control cells is diminished in the presence of ROCK inhibitor implicating a barrier-protective role for Rho kinases. Inhibition of the RhoA/ROCK pathway in PLXNA4 knockdown cells did not alter barrier formation nor barrier function of these endothelial cells. Localization of Rho kinase seems to be of great importance for its effect on endothelial barriers. Rho kinase localized at the cell margins, for example, has a barrier-protective activity, while it has barrier-disruptive activity when localized at F-actin stress fibers (47). We therefore speculate that perhaps in our control cells Rho kinase activity is high at junctional site, protecting the barrier. Inhibition of Rho kinases lowers this activity and impairs the endothelial barrier. In endothelial cells with decreased expression of PLXNA4 more stress fibers are present and there is more Rho kinase localization at the stress fibers, deteriorating the endothelial barrier. Inhibition of Rho kinases will inhibit both junctional and stress-fiber related Rho kinases and will have no additional effect on the endothelial barrier. Taken together, our data shows that the activity of the RhoA pathway is altered in endothelial cells with reduced PLXNA4 levels, but is not solely accountable for the increased vascular permeability seen in these cells. More in depth investigation of the precise contribution of small GTPases in endothelial cells during homeostasis and inflammation and in particular in combination with the PLXNA4 receptor, is necessary to fully grasp the extent of their function. In addition, further characterization of PLXNA4 signaling pathways, PLXNA4 ligand and (co-)receptor interactions are of great interest and could open up a new field for specific therapeutic targeting to maintain vascular integrity during the initial stages of atherosclerosis and potential other inflammation-related diseases.

**Figure 7:**
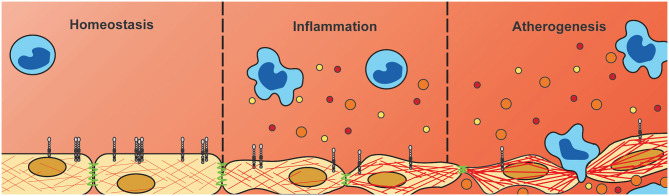
Downregulation of endothelial PLXNA4 under inflammatory conditions diminishes vascular integrity. Current hypothesis of the role of PLXNA4 in vascular integrity. Under homeostatic conditions PLXNA4 is expressed in endothelial cells resulting in a stable endothelial barrier. Inflammatory conditions reduce PLXNA4 expression resulting in rearranged cytoskeletal structures and cell-cell junctions. In turn this leads to decreased endothelial barrier function, impaired tube formation and increased vascular permeability to solutes and cells. This decrease in vascular integrity could contribute to atherogenesis by increasing deposition of (inflammatory) cells and lipids into the arterial wall.

In summary, we have shown differential expression of NGCs in both endothelial cells and monocytes under pro-inflammatory conditions, with most strikingly a consistent downregulation in endothelial PLXNA4 expression. Moreover, we have shown that PLXNA4, amongst others, is important for endothelial barrier function and that loss of PLXNA4 increases vascular leakage of solutes as well as (inflammatory) cells (Figure 7). Further investigation is required to gain a better insight into the mechanistic role of endothelial PLXNA4 in atherosclerosis and to elucidate potential therapeutic interventions.

## Data Availability Statement

The original contributions generated for this study are included in the article/Supplementary Material, further inquiries can be directed to the corresponding author/s.

## Author Contributions

DV, CB, GH, and JG conceptualized and designed the study. DV, CB, WS, SC, and ZN performed the data collection. DV, CB, RP, HZ, and JG contributed to the analysis and interpretation of the data. DV and CB drafted the paper. AZ, GH, and JG revised and approved the final version of the manuscript. All authors contributed to the article and approved the submitted version.

## Conflict of Interest

GH has served as consultant and speaker for biotech and pharmaceutical companies that develop molecules that influence lipoprotein metabolism, including Regeneron, Pfizer, MSD, Sanofi, and Amgen. Until April 2019, GH has served as PI for clinical trials conducted with A.O. Amgen, Sanofi, Eli Lilly, Novartis, Kowa, Genzyme, Cerenis, Pfizer, Dezima, Astra Zeneca. The Department of Vascular Medicine receives the honoraria and investigator fees for sponsor studies/lectures for companies with approved lipid lowering therapy in the Netherlands. Since April 2019, GH is partly employed by Novo Nordisk and the AMC. GH has no active patents nor share or ownership of listed companies. The remaining authors declare that the research was conducted in the absence of any commercial or financial relationships that could be construed as a potential conflict of interest.

## References

[1] Libby P. Inflammation in atherosclerosis. Arterioscler Thromb Vasc Biol. (2012) 32:2045–51. 10.1161/ATVBAHA.108.179705 22895665 PMC3422754

[2] Sabatine MS Giugliano RP Keech AC Honarpour N Wiviott SD Murphy SA. Evolocumab and clinical outcomes in patients with cardiovascular disease. N Engl J Med. (2017) 376:1713–22. 10.1056/NEJMoa1615664 28304224

[3] Braunwald E. Shattuck lecture–cardiovascular medicine at the turn of the millennium: triumphs, concerns, and opportunities. N Engl J Med. (1997) 337:1360–9. 10.1056/NEJM199711063371906 9358131

[4] Ross R. Atherosclerosis is an inflammatory disease. Am Heart J. (1999) 138:S419–20. 10.1016/S0002-8703(99)70266-8 10539839

[5] Libby P. Interleukin-1 Beta as a target for atherosclerosis therapy: biological basis of CANTOS and beyond. J Am Coll Cardiol. (2017) 70:2278–89. 10.1016/j.jacc.2017.09.028 29073957 PMC5687846

[6] Urschel K Cicha I. TNF-α in the cardiovascular system: from physiology to therapy. Int J Interferon Cytokine Mediat Res. (2015) 7:9–25. 10.2147/IJICMR.S64894

[7] Ridker PM Everett BM Thuren T MacFadyen JG Chang WH Ballantyne C. Antiinflammatory therapy with canakinumab for atherosclerotic disease. N Engl J Med. (2017) 377:1119–31. 10.1056/NEJMoa1707914 28845751

[8] Melani M Weinstein BM. Common factors regulating patterning of the nervous and vascular systems^*^. Annu Rev Cell Dev Biol. (2010) 26:639–65. 10.1146/annurev.cellbio.093008.093324 19575651

[9] Zhang H Vreeken D Bruikman CS van Zonneveld AJ van Gils JM. Understanding netrins and semaphorins in mature endothelial cell biology. Pharmacol Res. (2018) 137:1–10. 10.1016/j.phrs.2018.09.015 30240825

[10] Vreeken D Zhang H van Zonneveld AJ van Gils JM. Ephs and ephrins in adult endothelial biology. Int J Mol Sci. (2020) 21:5623. 10.3390/ijms21165623 32781521 PMC7460586

[11] Ghosh S Vivar J Nelson CP Willenborg C Segrè AV Mäkinen VP. Systems genetics analysis of genome-wide association study reveals novel associations between key biological processes and coronary artery disease. Arterioscler Thromb Vasc Biol. (2015) 35:1712–22. 10.1161/ATVBAHA.115.305513 25977570 PMC4841833

[12] Harst Pvd Verweij N. Identification of 64 novel genetic loci provides an expanded view on the genetic architecture of coronary artery disease. Circ Res. (2018) 122:433–43. 10.1161/CIRCRESAHA.117.312086 29212778 PMC5805277

[13] van Gils JM Ramkhelawon B Fernandes L Stewart MC Guo L Seibert T. Endothelial expression of guidance cues in vessel wall homeostasis dysregulation under proatherosclerotic conditions. Arterioscler Thromb Vasc Biol. (2013) 33:911–9. 10.1161/ATVBAHA.112.301155 23430612 PMC3647028

[14] Wanschel A Seibert T Hewing B Ramkhelawon B Ray TD Gils JMv. Neuroimmune guidance cue Semaphorin 3E is expressed in atherosclerotic plaques and regulates macrophage retention. Arterioscler Thromb Vasc Biol. (2013) 33:886–93. 10.1161/ATVBAHA.112.300941 23430613 PMC3647027

[15] Hu S Liu Y You T Heath J Xu L Zheng X. Vascular Semaphorin 7A upregulation by disturbed flow promotes atherosclerosis through endothelial β1 integrin. Arterioscler Thromb Vasc Biol. (2018) 38:335–43. 10.1161/ATVBAHA.117.310491 29269512 PMC5785426

[16] Qin RR Song M Li YH Wang F Zhou HM Liu MH. Association of increased serum Sema3E with TRIB3 Q84R polymorphism and carotid atherosclerosis in metabolic syndrome. Ann Clin Lab Sci. (2017) 47:47–51. 28249916

[17] Fujimaki T Kato K Yokoi K Oguri M Yoshida T Watanabe S. Association of genetic variants in SEMA3F, CLEC16A, LAMA3, and PCSK2 with myocardial infarction in Japanese individuals. Atherosclerosis. (2010) 210:468–73. 10.1016/j.atherosclerosis.2009.11.050 20036365

[18] Pascoe HG Wang Y Zhang X. Structural mechanisms of plexin signaling. Prog Biophys Mol Biol. (2015) 118:161–8. 10.1016/j.pbiomolbio.2015.03.006 25824683 PMC4537802

[19] Alto LT Terman JR. Semaphorins and their signaling mechanisms. Methods Mol Biol. (2017) 1493:1–25. 10.1007/978-1-4939-6448-2_1 27787839 PMC5538787

[20] Worzfeld T Offermanns S. Semaphorins and plexins as therapeutic targets. Nat Rev Drug Discov. (2014) 13:603–21. 10.1038/nrd4337 25082288

[21] Hruz T Laule O Szabo G Wessendorp F Bleuler S Oertle L. Genevestigator v3: a reference expression database for the meta-analysis of transcriptomes. Adv Bioinformatics. (2008) 2008:420747. 10.1155/2008/420747 19956698 PMC2777001

[22] Vreeken D Bruikman CS Cox SML Zhang H Lalai R Koudijs A. EPH receptor B2 stimulates human monocyte adhesion and migration independently of its EphrinB ligands. J Leukoc Biol. (2020) 108:999–1011. 10.1002/JLB.2A0320-283RR 32337793 PMC7496365

[23] Fontijn R Hop C Brinkman HJ Slater R Westerveld A van Mourik JA. Maintenance of vascular endothelial cell-specific properties after immortalization with an amphotrophic replication-deficient retrovirus containing human papilloma virus 16 E6/E7 DNA. Exp Cell Res. (1995) 216:199–207. 10.1006/excr.1995.1025 7813621

[24] Pau G Fuchs F Sklyar O Boutros M Huber W. EBImage—an R package for image processing with applications to cellular phenotypes. Bioinformatics. (2010) 26:979–81. 10.1093/bioinformatics/btq046 20338898 PMC2844988

[25] Montesano R Orci L. Tumor-promoting phorbol esters induce angiogenesis in vitro. Cell. (1985) 42:469–77. 10.1016/0092-8674(85)90104-7 2411423

[26] Gálvez BG Matias-Román S Albar JP Sánchez-Madrid F Arroyo AG. Membrane Type 1-Matrix metalloproteinase is activated during migration of human endothelial cells and modulates endothelial motility and matrix remodeling^*^. J Biol Chem. (2001) 276:37491–500. 10.1074/jbc.M104094200 11448964

[27] Giaever I Keese CR. Micromotion of mammalian cells measured electrically. Proc Natl Acad Sci U S A. (1991) 88:7896–900. 10.1073/pnas.88.17.7896 1881923 PMC52411

[28] Szulcek R Bogaard HJ van Nieuw Amerongen GP. Electric cell-substrate impedance sensing for the quantification of endothelial proliferation, barrier function, and motility. J Vis Exp. (2014) 85:51300. 10.3791/51300 24747269 PMC4159052

[29] van Duinen V van den Heuvel A Trietsch SJ Lanz HL van Gils JM van Zonneveld AJ. 96 perfusable blood vessels to study vascular permeability *in vitro*. Sci Rep. (2017) 7:18071. 10.1038/s41598-017-14716-y 29273771 PMC5741747

[30] van Duinen V Stam W Borgdorff V Reijerkerk A Orlova V Vulto P. Standardized and scalable assay to study perfused 3D angiogenic sprouting of iPSC-derived endothelial cells *in vitro*. JoVE. (2019) 153:e59678. 10.3791/59678 31762444

[31] de Kreuk BJ Nethe M Fernandez-Borja M Anthony EC Hensbergen PJ Deelder AM. The F-BAR domain protein PACSIN2 associates with Rac1 and regulates cell spreading and migration. J Cell Sci. (2011) 124:2375–88. 10.1242/jcs.080630 21693584

[32] Price LS Langeslag M ten Klooster JP Hordijk PL Jalink K Collard JG. Calcium signaling regulates translocation and activation of Rac. J Biol Chem. (2003) 278:39413–21. 10.1074/jbc.M302083200 12888567

[33] Kumanogoh A Kikutani H. Immunological functions of the neuropilins and plexins as receptors for semaphorins. Nat Rev Immunol. (2013) 13:802–14. 10.1038/nri3545 24319778

[34] Wong AW Brickey WJ Taxman DJ van Deventer HW Reed W Gao JX. CIITA-regulated plexin-A1 affects T-cell-dendritic cell interactions. Nat Immunol. (2003) 4:891–8. 10.1038/ni960 12910265

[35] Choi YI Duke-Cohan JS Ahmed WB Handley MA Mann F Epstein JA. PlexinD1 glycoprotein controls migration of positively selected thymocytes into the medulla. Immunity. (2008) 29:888–98. 10.1016/j.immuni.2008.10.008 19027330 PMC2615553

[36] Meda C Molla F De Pizzol M Regano D Maione F Capano S. Semaphorin 4A exerts a proangiogenic effect by enhancing vascular endothelial growth factor-a expression in macrophages. J Immunol. (2012) 188:4081. 10.4049/jimmunol.1101435 22442441

[37] Chabbert-de Ponnat I Marie-Cardine A Pasterkamp RJ Schiavon V Tamagnone L Thomasset N. Soluble CD100 functions on human monocytes and immature dendritic cells require plexin C1 and plexin B1, respectively. Int Immunol. (2005) 17:439–47. 10.1093/intimm/dxh224 15746246

[38] Roney KE O'Connor BP Wen H Holl EK Guthrie EH Davis BK. Plexin-B2 negatively regulates macrophage motility, Rac, and Cdc42 activation. PLoS ONE. (2011) 6:e24795. 10.1371/journal.pone.0024795 21966369 PMC3179467

[39] Yamamoto M Suzuki K Okuno T Ogata T Takegahara N Takamatsu H. Plexin-A4 negatively regulates T lymphocyte responses. Int Immunol. (2008) 20:413–20. 10.1093/intimm/dxn006 18209113

[40] Wen H Lei Y Eun SY Ting JP. Plexin-A4-semaphorin 3A signaling is required for Toll-like receptor- and sepsis-induced cytokine storm. J Exp Med. (2010) 207:2943–57. 10.1084/jem.20101138 21098092 PMC3005237

[41] Kigel B Rabinowicz N Varshavsky A Kessler O Neufeld G. Plexin-A4 promotes tumor progression and tumor angiogenesis by enhancement of VEGF and bFGF signaling. Blood. (2011) 118:4285–96. 10.1182/blood-2011-03-341388 21832283

[42] Treps L Le Guelte A Gavard J. Emerging roles of Semaphorins in the regulation of epithelial and endothelial junctions. Tissue Barriers. (2013) 1:e23272. 10.4161/tisb.23272 24665374 PMC3879177

[43] Mundi S Massaro M Scoditti E Carluccio MA van Hinsbergh VWM Iruela-Arispe ML. Endothelial permeability, LDL deposition, and cardiovascular risk factors—a review. Cardiovasc Res. (2017) 114:35–52. 10.1093/cvr/cvx226 29228169 PMC7729208

[44] Zhou Y Gunput R-AF Pasterkamp RJ. Semaphorin signaling: progress made and promises ahead. Trends Biochem Sci. (2008) 33:161–70. 10.1016/j.tibs.2008.01.006 18374575

[45] Beckers CML van Hinsbergh VWM van Nieuw Amerongen GP. Driving Rho GTPase activity in endothelial cells regulates barrier integrity. Thromb Haemost. (2010) 103:40–55. 10.1160/TH09-06-0403 20062930

[46] Spindler V Schlegel N Waschke J. Role of GTPases in control of microvascular permeability. Cardiovasc Res. (2010) 87:243–53. 10.1093/cvr/cvq086 20299335

[47] Amerongen GPvN Beckers CML Achekar ID Zeeman S Musters RJP Hinsbergh VWMv. Involvement of rho kinase in endothelial barrier maintenance. Arterioscler Thromb Vasc Biol. (2007) 27:2332–9. 10.1161/ATVBAHA.107.152322 17761936

