# Supplementary material for: Downregulation of Endothelial Plexin A4 Under Inflammatory Conditions Impairs Vascular Integrity
Source: Front Cardiovasc Med. 2021 May 4;8:633609. doi: 10.3389/fcvm.2021.633609 (PMC8129156; doi:10.3389/fcvm.2021.633609)

frontiers

Supplementary Material

1 Supplementary tables

1.1 Supplementary table 1: Primer sequences

Gene	Forward Sequence (5' → 3')	Reverse Sequence (5' → 3')
ADORA2B	GGGCTTCTGCACTGACTTCT	CCCGTGACCAAACTTTTATACCTG
DSCAM	ATCAGACCCAGCGAACTCAG	TGCAGCGGTAGTTATACAATCCA
EFNA1	TCAGGCCCATGACAATCCAC	GTGACCGATGCTATGTAGAACC
EFNA3	ACTGGAACAGCTCCAACCAG	AGTGCGGGCAGTAAATATCCA
EFNA4	AGATTCAGCGCTTCACACCC	GCCAGAACTCTCTGGAGTGG
EFNA5	ACTCTCCAAATGGACCGCTG	GCTTTAGACAGGACCTTCTTCCA
EFNB1	AGCTTCAGTAGTAGGACCGTC	AACTGTGCCAAACCAGACCA
EFNB2	TGTGGGTATAGTACCAGTCTTG	ACTGCTGGGGTGTTTTGATGG
EFNB3	TCGGCGAATAAGAGGTTCCA	GTCCCCGATCTGAGGGTACA
EPHA2	TCACACACCCGTATGGCAAA	ACGTTGCACACGGAGTACAT
EPHA4	TTCGCCCTATTTTCGTGTCTC	TGGTAGGTTCGGATTGGTGTAT
EPHA8	GCGCGTCTATGCTGAGATCAA	CGGTCCGACTCCAGGTAGT
EPHA10	CCCCGCAAAATCGACACGA	CGCGGCACTGCTTGTAGTA
EPHB2	GCTTCGAGGCCGTTGAGAAT	GAAGTGGTCCGGCTGTTGAT
EPHB3	GTCATCGCTATCGTCTGCCT	AAACTCCCGAACAGCCTCATT
EPHB4	CGCACCTACGAAGTGTGTGA	GTCCGCATCGCTCTCATAGTA
EPHB6	CGACCAGACCAATGGGAACA	GGGTGAAGGAGTGGGATTCG
GAPDH	CCTGCACCACCAACTGCTTA	GGCCATCCACAGTCTTCTGAG
NRP1	GGCGCTTTTCGCAACGATAAA	TCGCATTTTTCACTTGGGTGAT
NRP2	GCTGGCTATATCACCTCTCCC	TCTCGATTTCAAAGTGAGGGTTG
NTN4	GTACTTTGCGACTAACTGCTCC	TCCAGTGCATGGAAAAGGACT
NTN5	GGACCCATGCTACGATCCAC	AAGCGCAAGCTGACAGATGT
NTNG2	GCGCCTGAAGGACTACGTC	CGTTGCTGCATAGGTAGGGAT
PLXNA1	CGTGCTGTTCACTGTGTTCG	ACTGGATGCGCTCCTTAATCT
PLXNA2	CTACCTGTCCAGTGTCAACAAG	GGTCGGGAAGTAATCCTGCTT
PLXNA3	CGGACATGTTCAGTCTCGTGTA	CGCTGACGAAGCCGTAGAT

PLXNA4	GTCATTTGTCACATTCCGAGGA	GCTTGTAAATCCGATTGACGGC
PLXNB1	ACCAACTGCATTCACTCCCAA	GCACTCATCAGGCATCACAG
PLXNB2	CGTCCTACCAGTACCCCTTCT	TCACATCTGTCTCGTGGTTCAT
PLXNC1	CTACAAACTCGTTCCTGATCCTG	GTGGCTGTTAAACACTCCGAA
PLXND1	CTGTGCATGTGGAGTGATGG	GTTCCTTCCTCGGATGGTCAG
ROBO1	TAGTTCTTCGGACGGCTCCT	ACGCATGAAAATGTCGACGG
ROBO3	GTAGGACCGGAGGACGCTAT	CCCCGTTCTTGTACCACTCA
SEMA3A	CTTGCATTCATCTCTTCTGGTGT	GTGCCAAGGCTGAAATTATCCT
SEMA3C	GTATGTCTGTGGGAGTGGCG	ACGTTGGGGTTGAAAGAGCA
SEMA3F	CCACAGCGCATCGAGGAAT	CATGGGGTTGTAGGCACCTG
SEMA3G	TGGCTCGAACCATGTCACTG	CATTTGTTCACCAGCACCCG
SEMA4A	CTCCCCACATCTACGCAGTC	AGAGAGAAGGCACAAACCGC
SEMA4B	GAGCGGCCATTCCTCAGATTC	CACCCACGTACAGGGTCCT
SEMA4C	TTGTGCCGCGTAAGACAGTG	CCGTCAGCGTCAGTGTCAG
SEMA4D	GACAGCGATGGCATTTGCAC	CCTCCCGGGCACCTATGTA
SEMA4F	TCCAATCTCTGAGGCTGACTC	GGGATAAAGCGAAGATGGTGTC
SEMA5B	CGTGGCAGCCTATGATATTGG	AGCTCGTTATAGTAGAAGGGGAC
SEMA6A	AATCAGTATTTCGCATGGCAACT	GCAATGTAGAGGGTTCCGTTCA
SEMA6B	AAGGTGCTGACGACCTCAAC	CCTTCATCCGACACACGTTTATG
SEMA6C	ACAGTCATGTTCCTTGGCTCC	TGTTTGGGCTGTCCGCTTC
SEMA6D	GCTTTGTGCCTACATACTGCT	ACCGGATATTGCCTTGAATAGTG
SEMA7A	TTCAGCCCGGACGAGAACT	GAACCGAGGGATCTTCCCAT
SLIT2	AGCTTAGACGAATTGACCTGAGC	CCGAAGGCAGTTTATCTTGTTGG
SLIT3	CCTCTCTCGATGACGCTGAC	CGCCTTGACCTGGACAGAAAT
UNC5B	GCAGGACTCCACCACAAAAT	GCGAAATCGCTAGGGTATGTG
IL6	AAGCCAGAGCTGTGCAGATGAGTA	AACAACAATCTGAGGTGCCCATGC
VE-cadherin	GCACCAGTTTGGCCAATATA	GGGTTTTTGCATAATAAGCAGG
ICAM-1	GGCCGGCCAGCTTATACAC	TAGACACTTGAGCTCGGGCA
ITGB1	AACCGTAGCAAAGGAACAGCA	GCTCCCCTGATCTTAATCGCA

1.2 Supplementary table 2: Genevestigator NGC selection in monocytes

Gene	Mean	SE	Gene	Mean	SE
**PLXNB2**	14,877	0,06	**EFNA3**	10,689	0,032
**PLXNC1**	14,821	0,061	**PLXNA2**	10,603	0,032
**SEMA4A**	14,047	0,04	**ROBO3**	10,446	0,032
**SEMA4D**	13,736	0,055	**EFNA4**	10,431	0,049
**PLXND1**	13,587	0,051	**EPHB6**	10,367	0,051
**SEMA4B**	12,229	0,054	**EPHA10**	10,272	0,031
**SEMA3F**	12,132	0,058	**PLXNA1**	10,251	0,056
**SEMA5B**	11,994	0,043	**SLIT3**	10,234	0,026
**SEMA3C**	11,783	0,077	**EPHA8**	10,229	0,043
**SEMA4C**	11,494	0,055	**EFNB1**	10,163	0,032
**ADORA2B**	11,454	0,096	**PLXNB1**	10,139	0,026
**SEMA6B**	11,401	0,063	**NTNG2**	10,046	0,066
**EPHB2**	11,355	0,038	**NTN5**	10,045	0,037
**SEMA7A**	11,302	0,037	**NRP1**	10,035	0,136
**PLXNA3**	11,253	0,04	**DSCAM**	10,015	0,037
**SEMA6C**	11,231	0,047	**NRP2**	9,833	0,106
**EPHB3**	10,906	0,03	**SEMA3A**	9,14	0,044
**EPHB4**	10,754	0,035	**ROBO1**	8,265	0,041

1.3 Supplementary table 3: Genevestigator NGC selection in endothelial cells

Gene	Mean	SE	Gene	Mean	SE
**PLXND1**	15,148	0,046	**ROBO1**	13,145	0,090
**EFNB2**	14,832	0,083	**SEMA4C**	13,066	0,059
**NTN4**	14,813	0,120	**SLIT2**	12,876	0,099
**EPHB4**	14,548	0,054	**PLXNA1**	12,370	0,084
**SEMA3F**	14,460	0,073	**EPHB2**	12,198	0,051
**EFNA1**	14,261	0,093	**EFNA4**	12,068	0,065
**NRP1**	14,250	0,130	**EPHA4**	11,636	0,093
**PLXNA2**	14,203	0,082	**SEMA6B**	11,621	0,045
**EPHA2**	13,751	0,055	**ADORA2B**	11,604	0,062
**PLXNB2**	13,305	0,055	**SEMA3G**	11,460	0,108
**NRP2**	13,151	0,149	**EFNA5**	11,403	0,090
**ROBO3**	11,310	0,059	**SLIT3**	10,611	0,052
**SEMA6C**	11,298	0,031	**EPHB3**	10,545	0,041
**PLXNB1**	11,180	0,046	**EFNB1**	10,523	0,048
**SEMA4B**	11,171	0,057	**SEMA5B**	10,454	0,078
**SEMA4F**	11,146	0,061	**EFNB3**	10,261	0,056
**SEMA6D**	11,086	0,106	**UNC5B**	10,209	0,083
**PLXNA3**	10,910	0,034	**EPHA10**	10,182	0,027
**SEMA7A**	10,816	0,054	**SEMA4D**	10,130	0,053
**EFNA3**	10,754	0,042	**SEMA6A**	10,113	0,065
**PLXNA4**	10,670	0,079	**EPHA8**	10,063	0,034
**SEMA4A**	10,637	0,050	**SEMA3A**	10,021	0,088

2 Supplementary figures

2.1 Supplementary figure 1: Monocytic expression of NGCs under pro-atherogenic conditions.

Supplementary figure 1: Monocytic expression of NGCs under pro-atherogenic conditions. (**A, B**) Volcano plots depicting up and down regulation of NGCs after stimulation of freshly isolated human monocytes with IL1β (20 ng/ml) or TNFα (10ng/ml) for (A) 5 or (B) 24 hours. Results are depicted as mean of the natural logarithm transformed fold change in expression compared to unstimulated cells and plotted against significance. N=5.

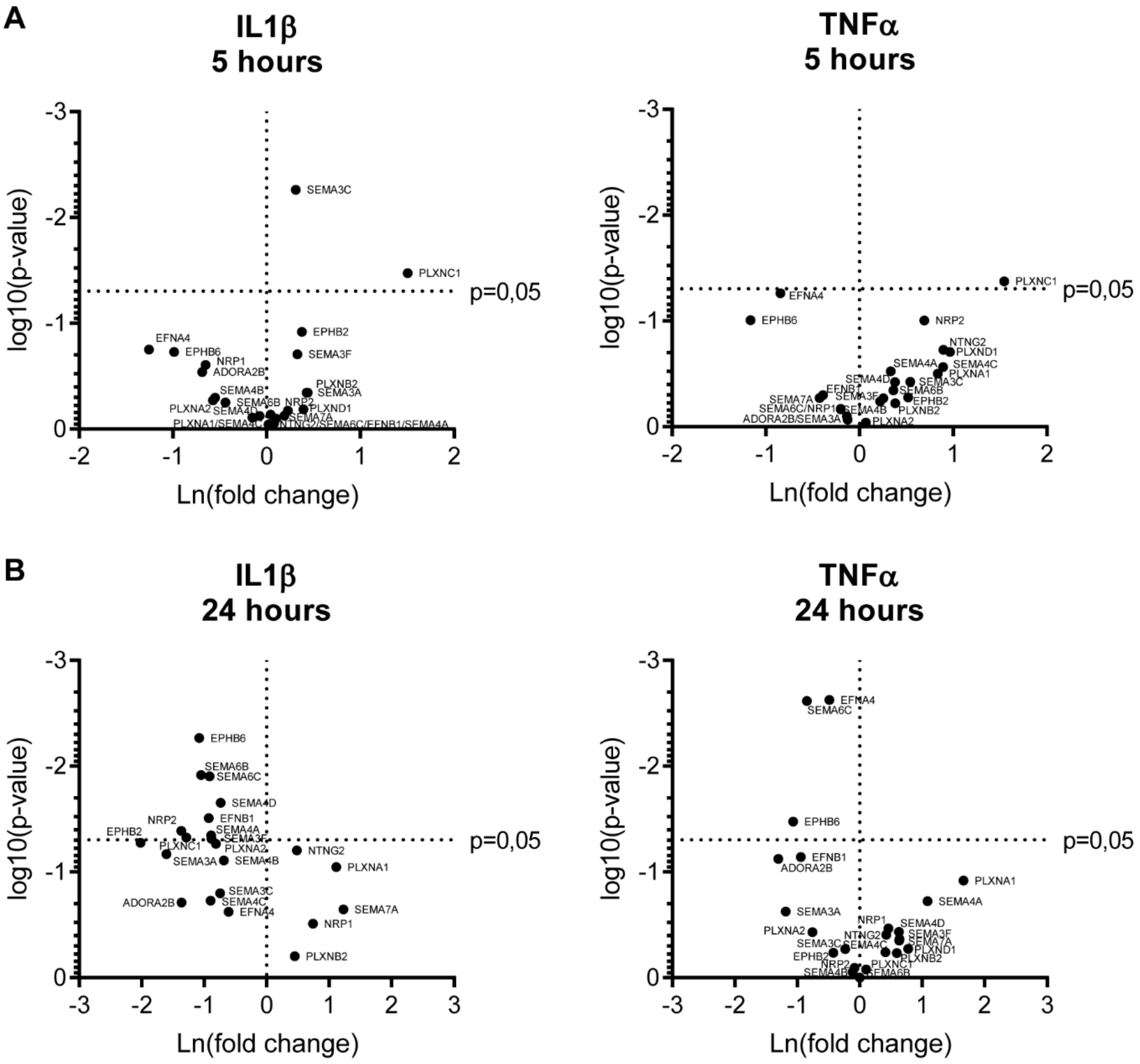

2.2 Supplementary figure 2: No effect of PLXNA4 overexpression on cellular morphology. (A) mRNA expression of PLXNA4, PLXNA1, PLXNA2 and PLXNA3 in immortalized endothelial cells treated with PLXNA4 overexpressing lentiviral particles (PLXNA4 OE) or control lentiviral particles (mock). Results are expressed as copies corrected for GAPDH. Mean ± S.E.M. of N=3, * P<0.05. (B) Representative overview pictures of control and PLXNA4 overexpression endothelial cells. (C) Representative fluorescent overview photographs of control and PLXNA4 overexpression endothelial cells stained for F-actin (red), VE-cadherin (green) and nuclei (blue). (D-E) Quantification of (D) VE-cadherin or (E) F-actin fluorescent signal in control and PLXNA4 overexpression endothelial cells. Results are expressed as fold change of (area*mean intensity)/nuclei relative to control cells, set at 1. Mean ± S.E.M. of N=3.

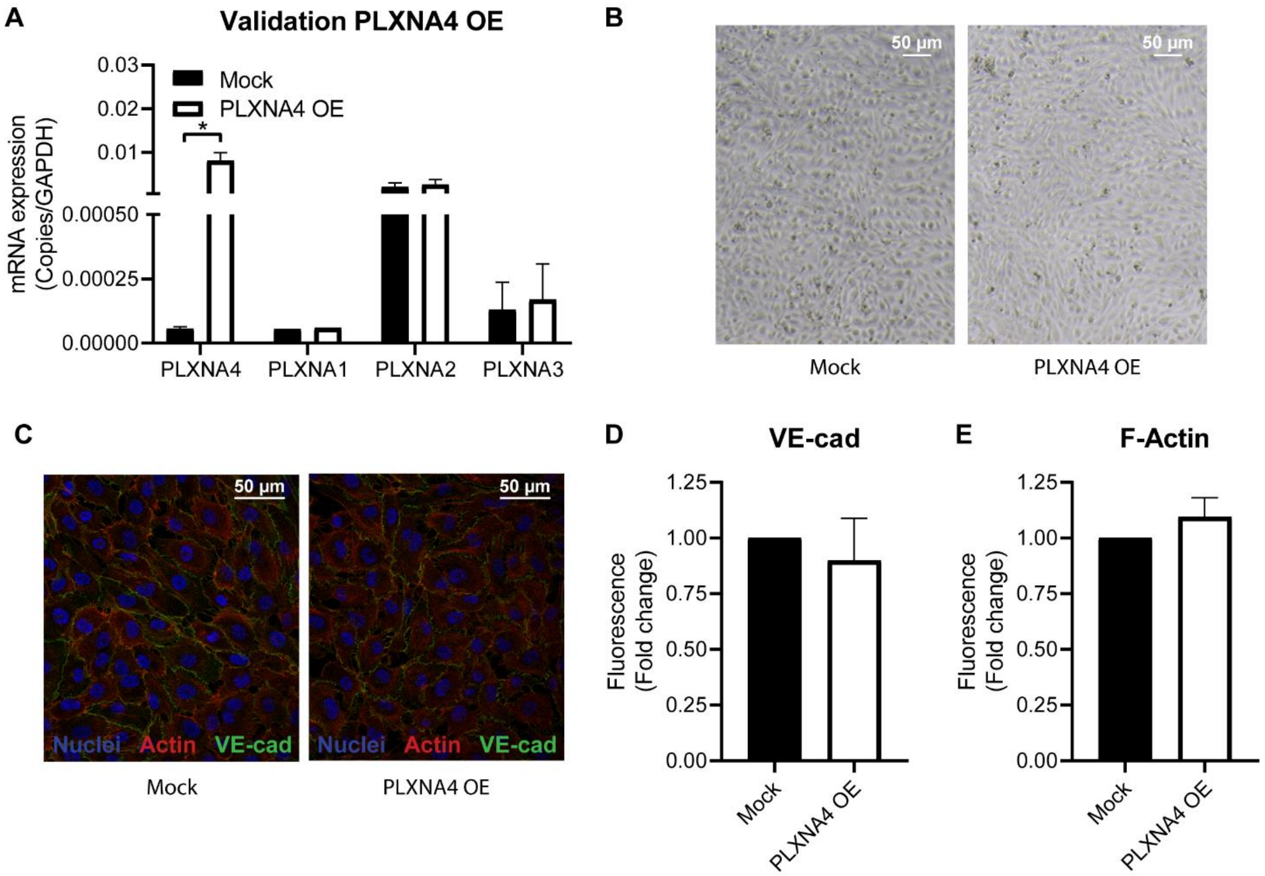

2.3 Supplementary figure 3: No effect of PLXNA4 overexpression on cell proliferation, migration and barrier function. (A) Proliferation of mock and PLXNA4 overexpression endothelial cells. Results are expressed as fold change to day 0. Mean ± S.E.M. of N=4. (B) Representative overview photographs and (C) quantification of migration of mock and PLXNA4 overexpression endothelial cells over time. Results are presented as percentage of open area. Mean ± S.E.M. of N=3. (D) Trans-endothelial electrical resistance of mock and PLXNA4 overexpression endothelial cells over time. Mean ± S.E.M. of N=3. (E-F) Endothelial electrical resistance attributable to (E) cell-cell contacts and (F) cell matrix contacts in monolayer of mock and PLXNA4 overexpression endothelial cells. Mean ± S.E.M. of N=3.

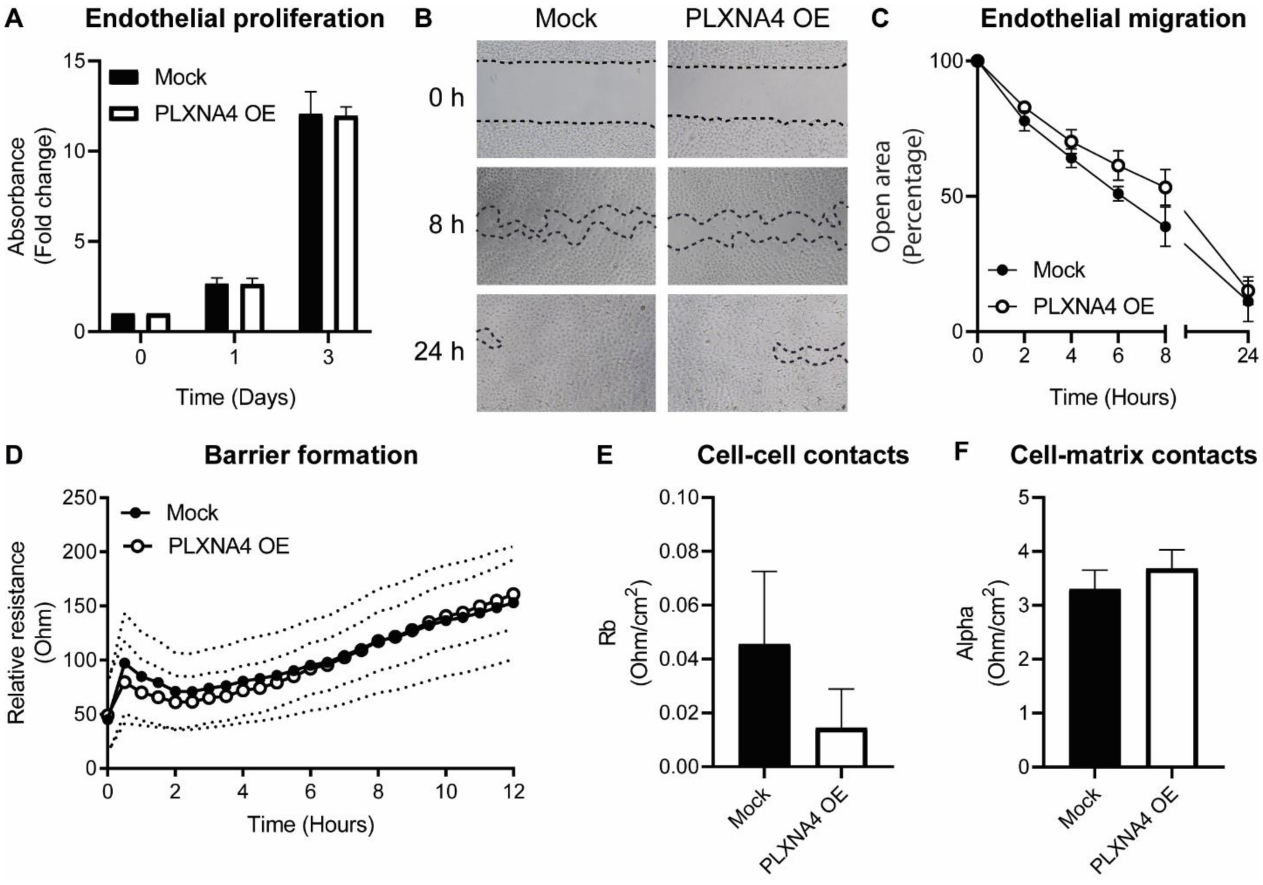

Supplement: Supplementary file 1 [file Data_Sheet_1.PDF]
